# Anti-PD-1-Induced Hidradenitis Suppurativa

**DOI:** 10.3390/dermatopathology8010007

**Published:** 2021-02-25

**Authors:** Alexia Maillard, Damien Pastor, Rastine Merat

**Affiliations:** Dermato-Oncology Unit, Division of Dermatology, University Hospital of Geneva, 1205 Geneva, Switzerland; alexia.maillard@etu.unige.ch (A.M.); damien.pastor@hcuge.ch (D.P.)

**Keywords:** anti-PD-1, cutaneous immune-related adverse events, hidradenitis suppurativa, melanoma

## Abstract

Mucocutaneous adverse events are commonly observed under immune checkpoint inhibitors (ICIs) therapy. Here, we report the case of a 43-year-old male patient with a stage IIIC melanoma disease who developed hidradenitis suppurativa (HS) three months after the beginning of an anti-PD-1 (nivolumab) adjuvant therapy. The patient had no comorbidities other than obesity and severe acne during adolescence. After an unsuccessful course of lymecycline while he was still treated with nivolumab, he gradually improved under zinc gluconate therapy and, more importantly, after nivolumab cessation. HS is a recurrent follicular inflammatory disease in the apocrine gland-bearing areas of the body often associated with obesity, metabolic syndrome, tobacco smoking, inflammatory bowel diseases, psoriasis, and arthritis. In our patient, the latency period between drug initiation and onset of HS symptoms and the improvement after immunotherapy discontinuation, argued strongly in favor of an anti-PD-1-induced HS. Anti-PD-1 therapies often trigger T cells-mediated adverse events that mimic Th17-mediated inflammatory and neutrophilic diseases. We suggest that HS, as other pustular skin reactions and ICIs-induced neutrophilic colitis, can be part of the anti-PD-1 mucocutaneous adverse event spectrum.

## 1. Introduction

Immune checkpoint inhibitors (ICIs) are increasingly used as a first line therapy in various malignancies. The use of monoclonal antibodies against cytotoxic T-lymphocyte-associated protein-4 (CTLA-4) and programmed cell death protein-1 receptor (PD-1) has dramatically improved patients’ outcome in melanoma disease including when used in the adjuvant setting in stage III disease. However, immune activation may lead to immune-related adverse events (irAEs), among which cutaneous irAEs (irCAEs) are by far the most frequent [1,2]. A broad spectrum of irCAEs has been described, the most frequent being pruritus, maculopapular rashes, lichenoid eruptions, and vitiligo. However, less common inflammatory and auto-immune cutaneous reactions in the spectrum of neutrophilic reactions, which include de novo or worsening of pre-existing psoriasis, have also been reported [3]. Here, we present a patient who developed hidradenitis suppurativa (HS) during anti-PD-1 nivolumab therapy.

## 2. Case Report

**Figure 1:**
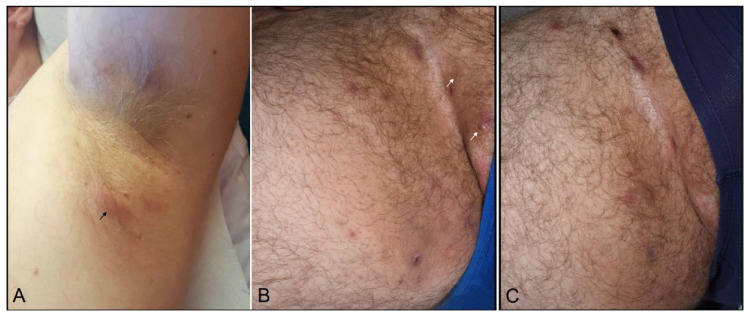
HS palpable nodules and aseptic abscess still occurring in the axillary folds (**A**) and the groins (**B**) four months after anti-PD-1 therapy discontinuation (arrows). (**C**) Remaining surgical drainage-induced scars ten months after adjuvant therapy discontinuation.

A 43-years-old non-smoking man, being obese with a BMI of 31.9 kg/m^2^, was diagnosed with a stage IIIC (pT3bpN1a(sn)M0) *BRAF*-wild type melanoma disease. He received a 3 mg/kg nivolumab adjuvant therapy every two weeks for 12 months (26 cycles). Three months after the beginning of the infusions, he developed painful nodules and abscesses of the axillary folds and groins. Most lesions cleared or drained spontaneously but there were new flares every week, requiring sometimes surgical drainage. The patient had a history of severe acne during puberty. He had no personal/family history of HS and no other medical condition associated with metabolic syndrome. On examination, there were inflammatory nodules and plaques, abscesses, but no fistula nor scars (Figure 1A,B). Bacterial culture on pus swab from a fistulated abscess and folliculitis were positives for a *Streptococcus Agalactiae group B* and a *Staphylococcus Epidermidis*.

A diagnosis of nivolumab-HS, stage Hurley 1 was made. Following an unsuccessful three month-period lymecycline 300 mg q.d. therapy, he received a combination of doxycycline 100 mg b.i.d. and zinc gluconate 60 mg q.d. The doxycycline was discontinued after one week because of the occurrence of gastro-intestinal symptoms. The patient was finally maintained under zinc gluconate alone with an excellent outcome (Figure 1C). Eighteen months after nivolumab cessation, he is finally cleared of HS lesions and remains in remission for melanoma disease.

## 3. Discussion

HS is a common recurrent follicular inflammatory skin disease that occurs in the apocrine gland-bearing areas of the body. It is often associated with obesity, metabolic syndrome, tobacco smoking, inflammatory bowel diseases, psoriasis, and inflammatory arthritis [4,5]. Even though HS is a clinically made diagnosis and biopsies are not routinely performed, the histopathological features of HS have been well characterized [6] and include, at the initial stage of the disease, an inflammation primarily made of neutrophils originating in the follicle and intraluminally within the apocrine, and sometimes the eccrine glands. This inflammation may lead to abscess formation surrounded by pyogenic and granulation reactions as well as to epidermal cyst and sinus tract formation and diffuse fibrosis.

De novo or worsening of other neutrophilic skin diseases as pre-existing psoriasis, and conditions primarily neutrophilic, i.e., acute generalized exanthematous pustulosis and Sweet syndrome have been reported in patients under ICIs [3,7]. Overall, the usually observed mean latency period between drug initiation and onset of irCAEs depends on the type of reaction and has been estimated to be 3–6 weeks for maculopapular rashes and as long as 6–12 weeks for lichenoid eruptions [1]. Regarding our patient, the time to onset was 12 weeks. Importantly, in addition to this temporal association, the improvement upon cessation of the drug and the absence of relevant personal and/or family history of HS, nor others risk factors except obesity, highly suggest that our patient’s HS was triggered by nivolumab.

Anti-PD-1 therapy removes the immune system inhibition allowing the control of tumor progression, but also the development of T cell-mediated adverse events that may be Th17-mediated, leading to the recruitment of neutrophils in tissues [8]. More importantly, IL-17 is a key cytokine in HS pathogenesis, TH17 cells being increased in lesional and perilesional HS skin [9]. The IL-17 axis is similarly critically involved in the pathogenesis of ulcerative colitis [10] and has been implicated in ICIs-induced colitis and the neutrophilic infiltrate observed in this disease [8]. By analogy, it is therefore tempting to suggest that the IL17 axis could be involved in ICIs-induced HS. Regardless of this hypothesis, our case report indicates that HS, as for other neutrophilic/pustular skin eruptions, but also the neutrophilic response observed in ICIs-induced colitis, can be part of the ICIs mucocutaneous adverse event spectrum.
